# Gelatin-Alginate Complexes for EGF Encapsulation: Effects of H-Bonding and Electrostatic Interactions

**DOI:** 10.3390/pharmaceutics11100530

**Published:** 2019-10-14

**Authors:** Seonghee Jeong, ByungWook Kim, Hui-Chong Lau, Aeri Kim

**Affiliations:** College of Pharmacy, CHA University, Seongnam-si, Gyeonggi-do 463-400, Korea; dooblue@hanmail.net (S.J.); huichong.lau@gmail.com (H.-C.L.)

**Keywords:** epidermal growth factor (EGF), polyelectrolyte complex, coacervate, alginate, gelatin, electrostatic interactions, H-bonding interactions, diabetic foot ulcer

## Abstract

Gelatin Type A (GA) and sodium alginate (SA) complexes were explored to encapsulate epidermal growth factor (EGF), and thereby to circumvent its proteolytic degradation upon topical application to chronic wounds. Phase diagrams were constructed based on turbidity as a function of GA to SA ratio and pH. Various GA-SA mixtures were compared for polydispersity index, zeta potential, *Z*-average, and ATR-FTIR spectra. Trypsin digestion and human dermal fibroblast scratch wound assay were done to evaluate the effects of EGF encapsulation. The onset pH values for coacervation and precipitation were closer together in high molecular weight GA (HWGA)-SA reaction mixtures than in low molecular weight GA (LWGA)-SA, which was attributed to strong H-bonding interactions between HWGA and SA probed by ATR-FTIR. EGF incorporation in both HWGA-SA precipitates and LWGA-SA coacervates below the isoelectric point of EGF, but not above it, suggests the contribution of electrostatic interactions between EGF and SA. EGF encapsulated in LWGA-SA coacervates was effectively protected from trypsin digestion and showed better in vitro scratch wound activity compared to free EGF. LWGA-SA coacervates are suggested as a novel delivery system for topical application of EGF to chronic wounds.

## 1. Introduction

Limited efficacy of topically applied growth factors to chronic wounds is in part attributed to the elevated level of proteases in the wound sites [1,2,3,4]. One way to enhance the efficacy of topically applied growth factors would be to protect them from proteases by employing appropriate drug delivery systems such as coacervates. Complex coacervates are known for very low interfacial energy in aqueous solution, which enables the coacervate to encapsulate a variety of molecules including proteins [5]. Once protein drugs are encapsulated in the coacervate phase, they could be protected from the external environment and maintain their bioactivity [6,7,8,9,10,11,12]. Coacervation is a spontaneous formation of a dense liquid phase from interactions of complementary macromolecular species [13,14]. For complex coacervation to occur by electrostatic interactions, the reaction pH should be adjusted so that the net charges of cationic and anionic polyelectrolytes are neutralized to form a polymer-rich liquid phase [13,15,16]. In our previous study, coacervates composed of high molecular weight Type A gelatin (HWGA) and sodium alginate (SA) showed high encapsulation efficiency for bovine serum albumin (BSA), protecting BSA from trypsin digestion. However, encapsulation of epidermal growth factor (EGF) was not successful in the HWGA-SA coacervates [17]. The difference in the encapsulation efficiency between BSA and EGF in HWGA-SA coacervates was attributed to the different level of binding of BSA and EGF to HWGA; the surface plasmon resonance analysis revealed high affinity of BSA to HWGA, but no significant binding of EGF to HWGA was detected.

Herein, we hypothesized that the encapsulation efficiency of EGF in GA-SA coacervates would improve if we could bring the coacervation pH below the isoelectric point (pI) of EGF to derive electrostatic interactions between positively charged EGF and negatively charged SA. GA, derived from collagen, has a net positive charge at pH below its pI of 7–9 [18], and because of its biocompatibility and biodegradability, various biomedical applications such as encapsulation, wound dressing, and biomineralization have been reported [19,20,21,22,23]. SA is a highly negative polymer with many applications in the pharmaceutical industry [24,25] and biomedical devices such as wound dressings [26,27]. In the present study, the dependence of interactions between GA and SA on the molecular weight of GA, pH, and polymer ratios were examined. ATR-FTIR spectroscopy was done to elucidate the interactions between GA and SA. GA-SA complexes were characterized with respect to their physical appearance, size distribution, and EGF encapsulation efficiency. For selected EGF-encapsulating coacervates, trypsin digestion and human dermal fibroblast scratch wound assay were done to evaluate their potential as a novel therapeutic modality for chronic wounds such as diabetic foot ulcer.

## 2. Materials and Methods

### 2.1. Materials

Gel strength 300 gelatin Type A (high molecular weight, HWGA, average 100 kDa, pI ~9), gel strength 90–110 gelatin Type A (low molecular weight, LWGA, average 20–25 kDa, pI ~7), and trifluoroacetic acid (TFA) were purchased from Sigma-Aldrich (St. Louis, MO, USA). Sodium alginate (SA) and acetic acid were purchased from Dae Jung (Gyeonggi-do, Korea). Epidermal growth factor (EGF) was provided by Daewoong Pharmaceutical Co., Ltd. (Seoul, Korea). Fetal bovine serum (FBS) and 0.25% Trypsin-EDTA were purchased from Gibco (Paisley, UK). Human dermal fibroblast (HDF) was purchased from Lonza (Walkersville, MD, USA). Precision plus protein dual color standard, polyvinylidene fluoride (PVDF) membrane, 30% acrylamid/Bis solution (29:1), 10% sodium dodecyl sulfate (SDS) solution, ammonium persulfate (APS), stacking gel buffer, resolving buffer, 10× Tris/Glycine/SDS buffer, 10× Tris/Glycine buffer, 10× Tris buffered saline (TBS), and tetramethylethylenediamine (TEMED) were purchased from Bio-rad (Heracules, CA, USA). Other reagents used (and vendors) were as follows: Dulbecco’s phosphate buffered saline (DPBS) (Welgene, Gyeongsangbuk-do, Korea), Dulbecco Modified Eagle Medium (DMEM) (Hyclone, Logan, UT, USA), Cell counting kit-8 (CCK-8) (Dojindo, Kumamoto, Japan), Bovine serum albumin (BSA) (Millipore, Bedford, MA, USA), 0.2 μm membrane filter (PALL, Port Washington, NY, USA), bicinchoninic acid assay (BCA) assay kit and enhanced chemiluminescence (ECL) Western Blotting Substrate (Thermo scientific, Rockford, IL, USA), silver staining kit (Biosesang, Gyeonggi-do, Korea), human EGF antibody as primary antibody (R&D systems, Minneapolis, MN, USA), and rabbit anti-mouse HRP as secondary antibody (Abcam, Cambridge, UK). 

### 2.2. Construction of Phase Diagrams

Solutions of HWGA (1%, *w*/*w*), LWGA (1%, *w*/*w*), and SA (0.5%, *w*/*w*) were prepared in distilled water. They were then mixed at various ratios of GA to SA for both HWGA and LWGA. Reaction conditions to construct phase diagrams included the GA to SA ratios of 1:1, 1:0.8, and 1:0.4, at a total polymer concentration of 5 mg/g. Aliquots of acetic acid were added as a pH modifier and the reaction pH was measured with a pH meter (Thermo Scientific, Waltham, MA, USA). Sample turbidity at 450 nm was measured with a microplate reader (Molecular Devices, San Jose, CA, USA). The zeta potential, particle size, and polydispersity index (PDI) were measured with Zetasizer Nano-ZS (Malvern instruments, Worcester, UK) in triplicate. Their mean and standard deviation were calculated with Microsoft Excel 2013. The microscopic appearances of coacervates were observed with an inverted phase-contrast light microscope (Nikon Eclipse Ts2, Tokyo, Japan).

### 2.3. Attenuated Total Reflectance Fourier Transform Infrared Spectroscopy (ATR-FTIR)

ATR-FTIR spectroscopy of various samples was performed using IRAffinity-1 (Dong-il Shimadzu, Japan) in conjunction with MIRacle single reflection horizontal ATR accessory (PIKE Technologies, Madison, WI, USA). A few milligrams of powder sample or a small portion of freeze-dried samples was placed on the ATR accessary plate. The clamp (High-Pressure Clamp) was then pressed down until its pin fully engaged to ensure even and reproducible contact between the sample and the accessary plate. We used a High-Pressure Clamp with Swivel Tip attached to the press. A total of 100 scans were collected in the range of 4000–500 cm^–1^. The experiments were done in duplicate for qualitative evaluation without statistical analysis.

### 2.4. Encapsulation of EGF in GA-SA Coacervates (EGF-Coacervate)

For encapsulation of EGF in coacervates, an aliquot of EGF solution was added to the mixed solution of GA and SA described above. The final EGF concentration in the reaction mixture was 100 μg/g. Aliquots of acetic acid were added as a pH modifier and the final reaction pH was measured. Qualitative analysis of EGF in the supernatants and pellets were done for various reaction conditions of GA-SA containing EGF. HWGA-SA and LWGA-SA reaction mixtures were centrifuged at 15,000 *g* for 15 min two times at 4 °C to obtain clear supernatants. The supernatants and the pellets dissolved in the same volume of PBS as the supernatant were analyzed by 15% SDS-PAGE. Each sample (20 μL) was mixed with 5× sample buffer (Bio solution, Seoul, Korea) and denatured at 100 °C for 10 min. SDS-PAGE was run in two steps, first at 80 V for 30 min followed by running at 100 V for 100 min. Then, the protein in the acrylamide gel was transferred to PVDF membrane at 100 V for 90 min. After blocking with 5% BSA (in TBST buffer (0.05% tween20 in TBS buffer) for 1 h at room temperature (RT), primary antibody (Human EGF antibody) was bound overnight at 4 °C. The membrane was probed with horseradish peroxidase (HRP)-conjugated secondary antibody (Rabbit anti-mouse HRP) for 1 h at RT. Labeled protein band was reacted with ECL Western Blotting Substrate and detected by image quant LAS 4000 (GE Healthcare, Little Chalfont, UK).

For qualitative determination of EGF encapsulation and loading efficiency, coacervates reaction mixtures were centrifuged for 20 min at 15,000 *g* and the unencapsulated EGF in the supernatants was measured by HPLC. HPLC conditions were as follows: AZURA HPLC system (Knauer, Berlin, Germany), HPLC Column (Capcell pack C18, 4.6 × 150 mm, 3 µm, Shiseido), Mobile phase A: DW in 0.1% trifluoroacetic acid B: Acetonitrile in 0.1% trifluoroacetic acid (A:B = 85:15 at 0, 60:40 at 5, 50:50 at 7, 85:15 at 8, and 85:15 at 12 min), Flow: 1.5 mL/min, injection volume: 20 µL, wavelength: UV 280 nm, column temp. 20 °C.

Experiments were done in triplicate for each composition. Their means and standard deviations were calculated with Microsoft Excel 2013. Calculation of %encapsulation and %loading efficiency was done according to the equations below:

% Encapsulation efficiency = [(Total EGF – Free “unencapsulated EGF”)/Total EGF] × 100.

% Loading efficiency = [Encapsulated EGF/polymer complex dried weight] × 100.

### 2.5. Preparation of Freeze-Dried Samples and Electron Microscopy 

Physical mixtures of EGF, LWGA, and SA (EGF-PM) were prepared by simple mixing of each stock solution. Two compositions of EGF-coacervates were selected for freeze-dried samples, LWGA-SA (1:1) at pH 4.14 and LWGA-SA (1:0.4) at pH 4.34. The samples were freeze-dried in a freeze-dryer (Operon, Gyeonggi-do, Korea) after freezing overnight at −80 °C (Ultra-low temperature freezer, Sanyo, Osaka, Japan). The freeze-dried samples were kept at −20 °C in heat-sealed aluminum pouches until use. The vehicle control samples of physical mixtures and coacervates without EGF were also freeze-dried using the same method. Microscopic structures of freeze-dried samples were examined with a field emission scanning electron microscope (FE-SEM 7800F prime, JEOL Ltd., Tokyo, Japan) at the National Center for Inter-university Research Facilities at Seoul National University. Double-sided adhesive carbon tape was placed on labeled stainless steel stubs and the samples were placed on the exposed side of the carbon adhesive taking care not to damage the surface topography of the samples. Internal structures of the freeze-dried samples were examined after tearing off the surface of freeze-dried samples using a pincette. They were then coated with platinum (Cressington Sputter Coater 108) and placed in the chamber of the microscope. 

### 2.6. Trypsin Digestion Assay of EGF-Coacervate

The freeze-dried samples were placed in sample tubes and trypsin-EDTA (40 μg) was added. Control samples were 10 μg EGF with and without 40 μg Trypsin-EDTA. To adjust the pH of EGF-coacervate samples for optimal trypsin activity, 1 μL of 70 mM NaOH was added in the sample tubes. After 1 and 2 h incubation at 37 °C, 100 rpm, EGF remaining in the samples was measured by Western blot as described in Section 2.4.

### 2.7. Human Dermal Fibroblast (HDF) Scratch Wound Assay

To prepare gelatin-coated plates, 0.1% gelatin solution was added in 24-well plates after marking a mid-line on the back of each well with a marker pen. The plates were incubated at 37 °C for 2 h and then washed once with DPBS. HDFs at passage 7 were cultured in a 100π dish until 80% confluence. After the cells were detached using 0.25% trypsin-EDTA, 9 × 10^4^ cells were seeded per well in the gelatin-coated plate and incubated at 37 °C overnight. In vitro scratch wounds were then created by scratching the cells at the mid-line of each well with a 200 μL pipette tip and the wells were washed twice with DPBS to remove debris. A total of 1 mL medium containing 0.5% FBS was added to each well and pictures were taken with a light microscope (Leica, Wetzlar, Germany). For each LWGA-SA ratio of (1:1) and (1:0.4), four different freeze-dried samples were tested: (1) LWGA and SA in solution, (2) LWGA, SA, and EGF in solution, (3) coacervate of LWGA and SA without EGF, and (4) coacervate of LWGA and SA encapsulating EGF. Freeze-dried samples were put in the Transwell inserts and 200 μL medium containing 0.5% FBS in DMEM was added on them. The inserts were then placed in the wells containing 0.5% FBS in DMEM 800 μL. Culture media containing 0.5% FBS were used except for the following groups: 10% FBS in DMEM 1 mL (positive control), 0.5% FBS in DMEM 1 mL (negative control), and 10 ng/mL EGF in 0.5% FBS in DMEM 1 mL (EGF solution samples). After incubation at 37 °C for 8 h, microscopic images were analyzed using Image-Pro Plus software (Media Cybernetics, USA) to measure the migration of HDF cells filling the scratched area. One way ANOVA was done with GraphPad Prism software for statistical analysis. The results in triplicates were normalized to the negative control as follows:

Fold of wound area = {(*A*_0_−*A*_8_)/*A*_0_}_sample_/{(*A*_0_−*A*_8_)/*A*_0_}_negative_, (1)

where *A*_0_ is the original wound area and *A*_8_ is the wound area after 8 h.

## 3. Results

### 3.1. Phase Diagrams of HWGA-SA and LWGA-SA 

**Figure 1:**
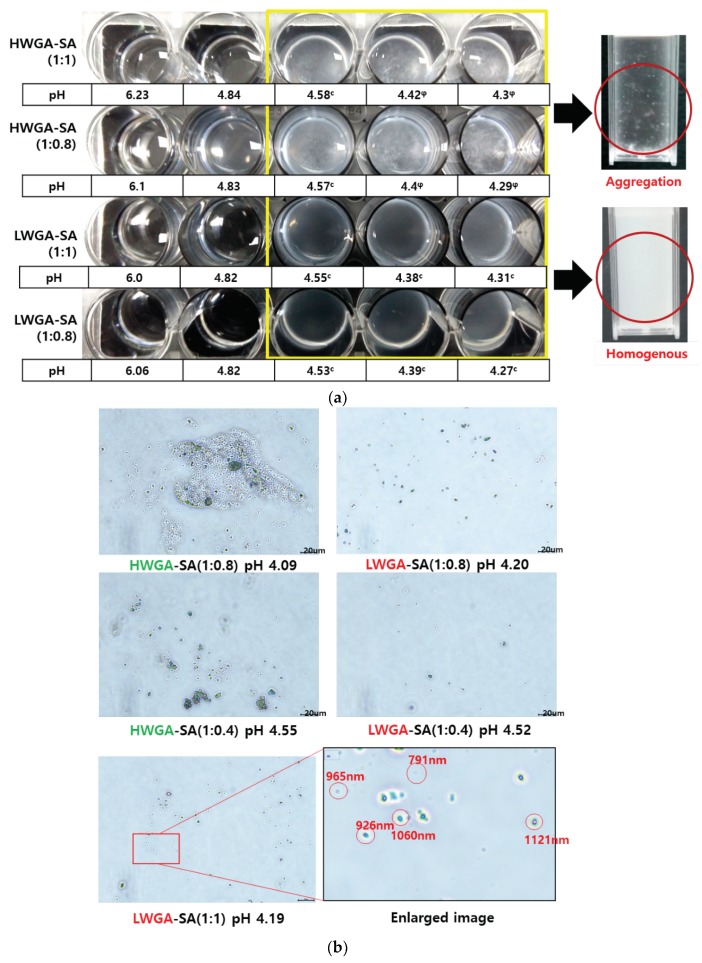

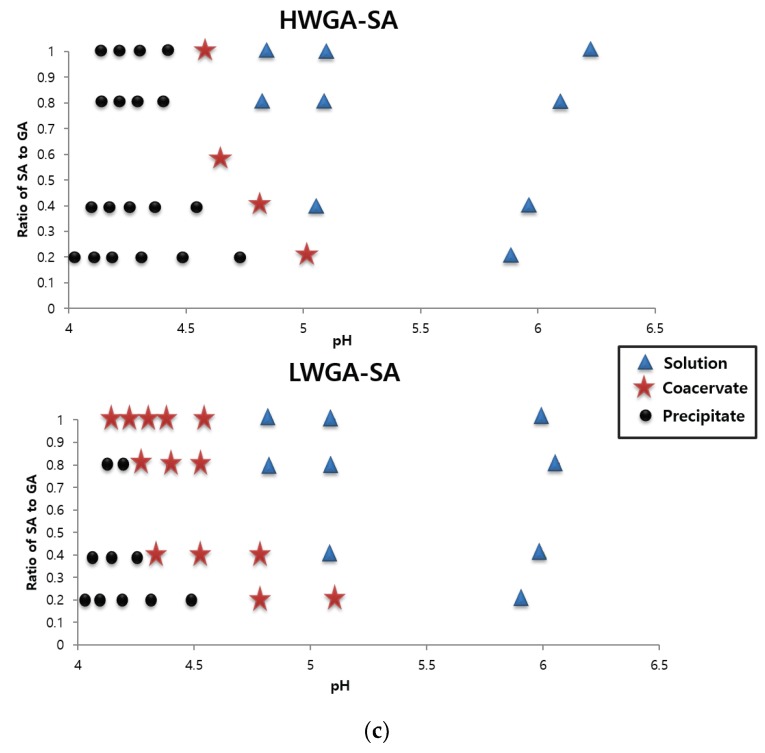
Comparison of complex phases among reaction conditions using high molecular weight gelatin Type A (HWGA) and low molecular weight GA (LWGA). (**a**) Macroscopic appearance of polymer complex phases of various polymer ratios and reaction pHs. (**b**) Representative images of phase contrast light microscopy to compare HWGA-sodium alginate (SA) and LWGA-SA. (Scale bars = 20 μm). (**c**) Phase diagrams of HWGA-SA and LWGA-SA as a function of pH and the SA to GA ratio.

Figure 1a compares the changes in the physical appearance of HWGA-SA and LWGA-SA upon pH titration at various polymer ratios. From GA-SA ratio of 1:1, HWGA-SA started to show precipitates at pH 4.42 and massive precipitation at lower pH, whereas LWGA-SA was homogenous down to pH 4.31. When these samples were compared under the phase-contrast light microscope, HWGA-SA appeared as aggregates whereas LWGA-SA was homogenous colloid (Figure 1b). These macroscopic and microscopic appearances of coacervates and precipitates are similar to those reported by others previously [15,28,29,30]. Figure 1c shows the phase diagrams obtained from visual observation and turbidity measurement of the reaction mixtures at various combinations of GA to SA ratios and the reaction pH. Turbidity has been frequently used to describe phase behaviors of polyelectrolyte complexes [28,31,32]. For the HWGA-SA ratio from 1:1 to 1:0.2, coacervation occurred in narrow pH ranges, followed by massive precipitation at lower pH. In contrast, LWGA and SA yielded liquid-coacervate in a broader range of GA to SA ratios and pH values. 

Coacervation requires sufficiently large charges on the polyelectrolytes to cause significant electrostatic interactions, but not so large to avoid precipitation [13]. The particular pH (pHc) required for coacervate formation varies depending on the molecular weight and the polymer ratios [13,31,32,33,34]. The pHφ, representing the pH of the first occurrence of a precipitate, also depends on the composition of reaction mixtures [13]. According to Comert et al., coacervation required complex charge neutralization, while precipitation, always preceded by coacervation, required intimate protein-polyanion contact [28]. They observed the pH interval between coacervation and precipitation depended on charge anisotropy of proteins. The narrow interval between the onset pH of coacervation and precipitation in HWGA-SA shown in Figure 1c suggests that intermolecular interactions are stronger in HWGA-SA than in LWGA-SA. FTIR spectra results are discussed below to elucidate molecular mechanisms for such difference. Dependence of coacervation on the polymer molecular weight was also reported by Chollakup, et al.; coacervation of polyacrylic acid and polyallylamine depended on the molecular weight of polyacrylic acid and the lower molecular weight had a wider range of coacervation reaction conditions [34].

Table 1 shows PDI, zeta potential, and Z-average diameters as a function of pH at the GA to SA ratio of 1:0.4. More data on the GA to SA ratios of 1:1 and 1:0.8 are provided in Supplementary Materials (Tables S1 and S2) The onset pH for coacervate formation, denoted as pHc, was determined from the abrupt increase in the sample turbidity and decrease in PDI as the reaction pH was lowered. The onset pH for precipitation (pHφ) was identified from the abrupt increase in *Z*-average and PDI due to massive aggregate formation upon further lowering of the pH.

Coacervation was associated with a decrease in PDI at all test conditions, suggesting that the polydispersity of gelatin and alginate molecules decreased upon the formation of homogenous colloidal phase between these molecules. Similar results have been reported previously for casein-gum arabic coacervates; gum arabic solution showed multiple peaks in particle size distribution whereas soluble complex coacervates showed a single peak at the same pH [31]. PDI values less than 0.2 are typically associated with a monodisperse system, while those higher than 0.7 indicate a highly polydisperse system [11]. Monodisperse coacervates bear the highest surface charge. As the surface charge approaches neutrality, the repulsion force between coacervates decreases and aggregates form more readily. 

The zeta potentials of HWGA-SA solutions (−51~−53 mV) without any acid addition were lower than those of LWGA-SA (−35~−42 mV) at all three ratios of GA-SA (Tables S1 and S2). Such difference cannot be explained by the difference in the net charges between HWGA and LWGA because their pI values are ~9 and ~7, respectively. It may suggest that HWGA carries less effective positive charge to interact with SA, due to its larger average molecular weight than LWGA. The difference in zeta potentials between HWGA-SA and LWGA-SA became less than 5 mV when the pH was lowered. For example, at pH 4.8, the zeta potentials of HWGA-SA and LWGA-SA were −26.8~−38.9 mV and −25~−36.8 mV, respectively. The Z average diameters of LWGA-SA increased upon the pH lowering while those of HWGA-SA decreased for 1:1 and 1:0.8 ratio of GA-SA. Taken together, the addition of acid to LWGA-SA might have induced association of LWGA molecules to carry a similar effective positive charge as HWGA. 

### 3.2. ATR-FTIR 

**Figure 2:**
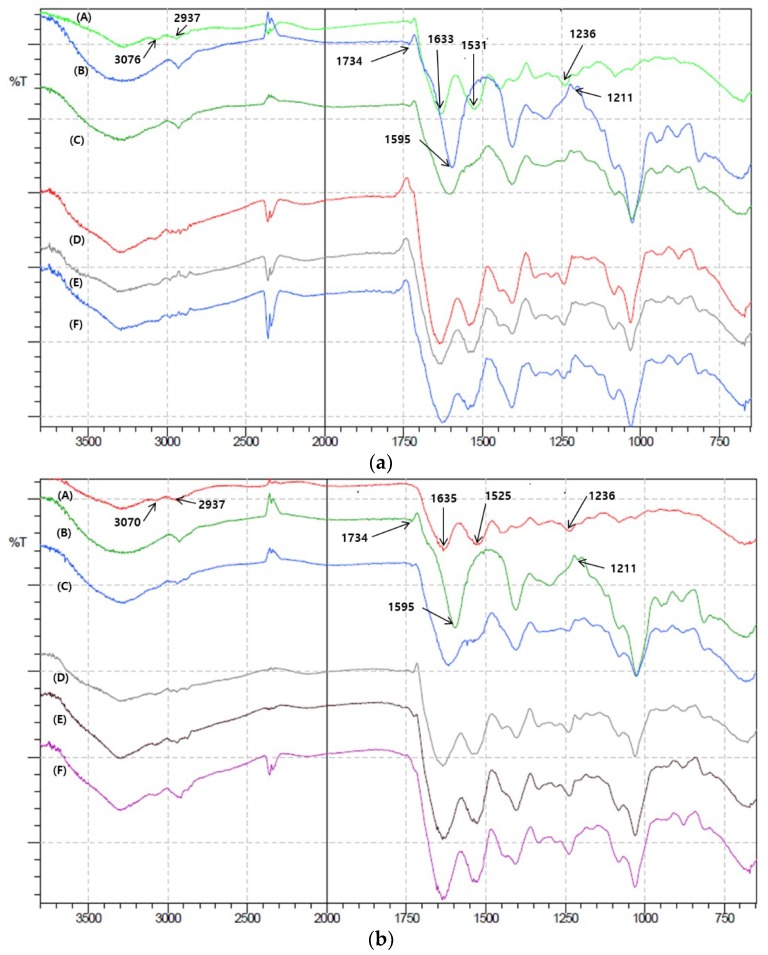
ATR-FTIR of various samples. (**a**) (A) Powder HWGA, (B) powder SA, (C) physical mixture of powder HWGA and SA (1:0.4), (D) freeze-dried HWGA-SA (1:0.4) solution, (E) freeze-dried HWGA-SA (1:0.4) coacervates, (F) freeze-dried HWGA-SA (1:0.4) precipitates. (**b**) (A) Powder LWGA, (B) powder SA, (C) physical mixture of powder LWGA and SA, (D) freeze-dried LWGA-SA (1:0.4) solution, (E) freeze-dried LWGA-SA (1:0.4) coacervates, (F) freeze-dried LWGA-SA (1:0.4) precipitates.

To delineate molecular mechanisms underlying different phase behaviors of HWGA and LWGA, ATR-FTIR of various samples were obtained; powder samples of (GA, SA, and their physical mixtures), and freeze-dried samples of (GA-SA solutions, coacervates, and precipitates). It would be ideal to perform FTIR in the liquid state prior to freeze-drying. However, we resorted to FTIR data obtained with freeze-dried samples because of low spectral quality of various samples in water. Previous studies by Gashti et al. and Li et al. also used solid-state samples to provide evidence of intermolecular interactions by FTIR [22,23,31]. Even in a freeze-dried state, we were able to find the differences among various samples to speculate different levels of H-bonding and electrostatic interactions depending on the gelatin molecular weight as shown in Figure 2a,b for HWGA and LWGA, respectively. Numerical summation of the spectra of GA and SA powder samples resulted in the same spectra as those of physical powder mixture samples shown in Figure 2, suggesting no particular interactions between powder samples (data not shown). Major bands in the FTIR spectra of GA and SA were assigned based on previous studies [22,23,31,35] and a textbook on spectroscopy [36].

The spectral assignments of major bands and their changes are summarized in Table 2 and Table 3 for HWGA and LWGA, respectively. A band in the spectra of GA at 3070~3076 cm^−1^ originating from NH3+ remained the same for all three GA-SA reaction conditions. Two major bands of SA are assigned to C=O stretching; one at 1595 cm^−1^ is assigned to anionic carboxylate and the other at 1734 cm^−1^ to carboxylic acid [35]. The peak at 1595 cm^−1^ disappeared in all three reaction conditions of GA-SA mixtures for both HWGA and LWGA, suggesting that electrostatic interactions between GA and SA occur even at pH 6.0 where they were still in solution. These findings are consistent with previous studies on ion-pairing or soluble complex formation between oppositely charged macromolecules prior to coacervation [25,36]. Changes in the carboxylic acid C=O stretching peak of SA at 1734 cm^−1^ differ between HWGA and LWGA. The peak disappeared in all three reaction conditions for HWGA-SA mixtures whereas it is visible for all three LWGA-SA reaction mixtures with a slight redshift from 1734 cm^−1^ to 1730 and 1724 cm^−1^. The C=O stretching vibration region is known to be useful to probe intermolecular H-bonding; disappearance of C=O stretching band around 1724 cm^−1^ due to intermolecular H-bonding between poly(3-hydroxybutyrate) and poly(4-vinylphenol), and changes in the carbonyl stretching band of methyl acetate upon H-bonding with solvent molecules were reported previously [37,38]. Likewise, the disappearance of C=O stretching at 1734 cm^−1^ of SA in HWGA-SA mixtures suggests H-bond interactions which are not apparent in LWGA-SA reaction mixtures. Therefore, H-bonding interactions could be an explanation for early onset of precipitation in HWGA-SA reaction mixtures at pH where LWGA-SA still forms coacervates without precipitation. Differences in the peak splitting in the bands assigned to amide II around 1525 cm^−1^ (LWGA) and 1531 cm^−1^ (HWGA) also suggest different intermolecular interactions with SA. A recent report by Iwashita et al. describes the effect of protein unfolding on coacervates formation between ovalbumin and lysozyme [39]. They suggested that electrostatic interactions play a major role in coacervate formation between oppositely charged proteins in the native state whereas hydrophobic interactions between unfolded proteins resulted in solid-like coaggregates. Notably, spectral patterns of C-H vibration around 2937 cm^−1^ show remarkable differences between HWGA-SA and LWGA-SA; the latter maintains the band pattern of the powder mixture while the former loses a distinct band observed in the spectrum of the powder mixture. This particular region cannot be assigned specifically to GA or SA because both molecules contain numerous C-H bonds with their bands overlapping. There were differences in C–O–C stretching of the pyranose ring too; for HWGA-SA, the peak disappeared starting at one phase solution sample whereas LWGA-SA solution sample showed a slight shift. We speculate from these observations that differences in overall interactions between HGWA-SA and LWGA-SA result in different structural environments even in one phase solution reaction. To the best of our knowledge, the present study is the first to report on different phase behaviors between high and low molecular weight gelatins in terms of their interaction with SA, in particular, possibility of H-bond interactions affecting the onset pH of precipitation.

### 3.3. Encapsulation of EGF in GA-SA Coacervate

**Figure 3:**
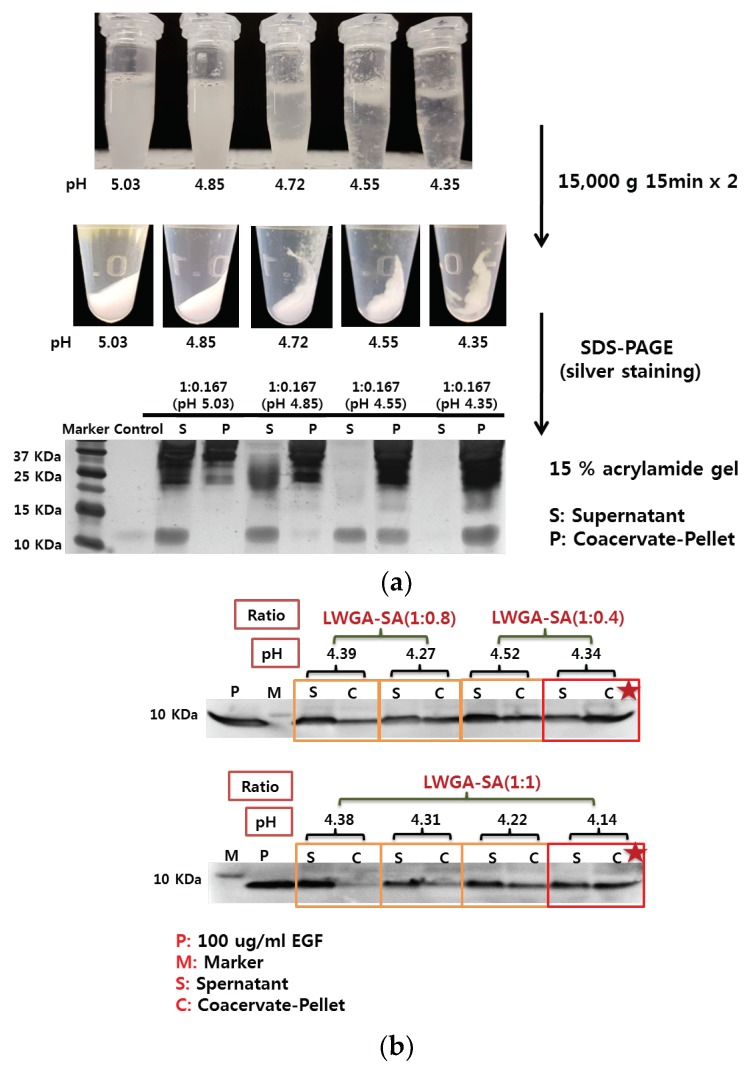
Qualitative analysis of epidermal growth factor (EGF) by Western blot in various GA-SA reaction mixtures. (**a**) Appearance of HWGA-SA (1:0.167) as a function of reaction pH, and EGF in the supernatants and pellets determined by Western blot after centrifugation. (**b**) EGF in the supernatants and pellets from eight different reaction conditions of LWGA-SA. Sample loading was 20 μL per well. P: EGF solution, M: marker, S: supernatant, P: pellet.

Encapsulation efficiency of EGF in liquid coacervates samples was measured instead of the final freeze-dried samples as done by Chu et al. and Lau et al. [6,18]: firstly, encapsulation in liquid coacervates could be readily determined after separation of dense phase from dilute phase by centrifugation and SDS-PAGE of each phase. Secondly, the freeze-dried samples return to liquid coacervate once hydrated after application and therefore it would be more relevant to see the effect of encapsulation in coacervate on protection from trypsin and in vitro activity. The reaction mixture of HWGA-SA (1:0.167) at pH 5.03 appeared as homogeneous colloids with all EGF associated with the supernatant (Figure 3a). At pH 4.85 they were still homogenous coacervates with a slightly visible EGF band in the pellet. As the reaction pH was lowered, precipitates appeared with more EGF in the pellet and all EGF appeared associated with the pellet at pH 4.35, demonstrating EGF incorporation in their massive precipitates at pH below the pI of EGF. The data suggest the electrostatic interactions between EGF and SA contribute to EGF incorporation in HWGA-SA precipitates. Although HWGA-SA precipitates effectively incorporate EGF, they are not a desirable drug delivery system because of their inhomogeneity and difficulty in handling. Among various compositions of coacervates shown in Tables S1 and S2, homogenous LWGA-SA coacervates with turbidity above 1.5 and PDI below 0.4 were selected to determine the encapsulation efficiency of EGF. Eight reaction conditions selected were LWGA-SA (1:1) with the final pH of 4.38~4.14, LWGA-SA (1:0.8) with the final pH of 4.39 and 4.27, and LWGA-SA (1:0.4) with the final pH of 4.52 and 4.34. Figure 3b shows the Western blot results of EGF in the supernatants and the pellets after centrifugation of various compositions of coacervates. Relative band intensities of the pellets at all three polymer ratios increased as the pH decreased suggesting improved EGF encapsulation efficiency at lower pH. Among all the tested compositions, the LWGA-SA (1:0.4) at pH 4.34 condition resulted in the highest EGF encapsulation efficiency. In our previous study, coacervates composed of several ratios of HWGA-SA (3:1, 4:1, 5:1, and 6:1) titrated to pH 5.0 effectively encapsulated BSA but not EGF [17]. Further optimization using various polymer ratios of either HWGA and SA or HWGA and hyaluronic acid, at various salt concentration did not improve the encapsulation efficiency of EGF. Such negative results with HWGA-SA or HWGA-hyaluronic acid system for EGF encapsulation were attributed to negligible binding interactions between EGF and HWGA. The higher encapsulation efficiency of EGF in LWGA-SA coacervates with the decrease in pH (Figure 3b) supports our hypothesis that the encapsulation efficiency of EGF in GA-SA coacervates would be improved at lower pH by introducing a more positive charge on EGF, thereby enhancing its electrostatic interactions with SA.

Quantitative encapsulation efficiency and loading efficiency determined by HPLC analysis of the supernatants are summarized in Table 4. Both encapsulation efficiency and loading efficiency were higher for GA:SA of 1:0.4 than that of 1:1. The results are consistent with the qualitative results shown by SDS-PAGE (Figure 3b). 

### 3.4. Fabrication of Freeze-Dried Sample

**Figure 4:**
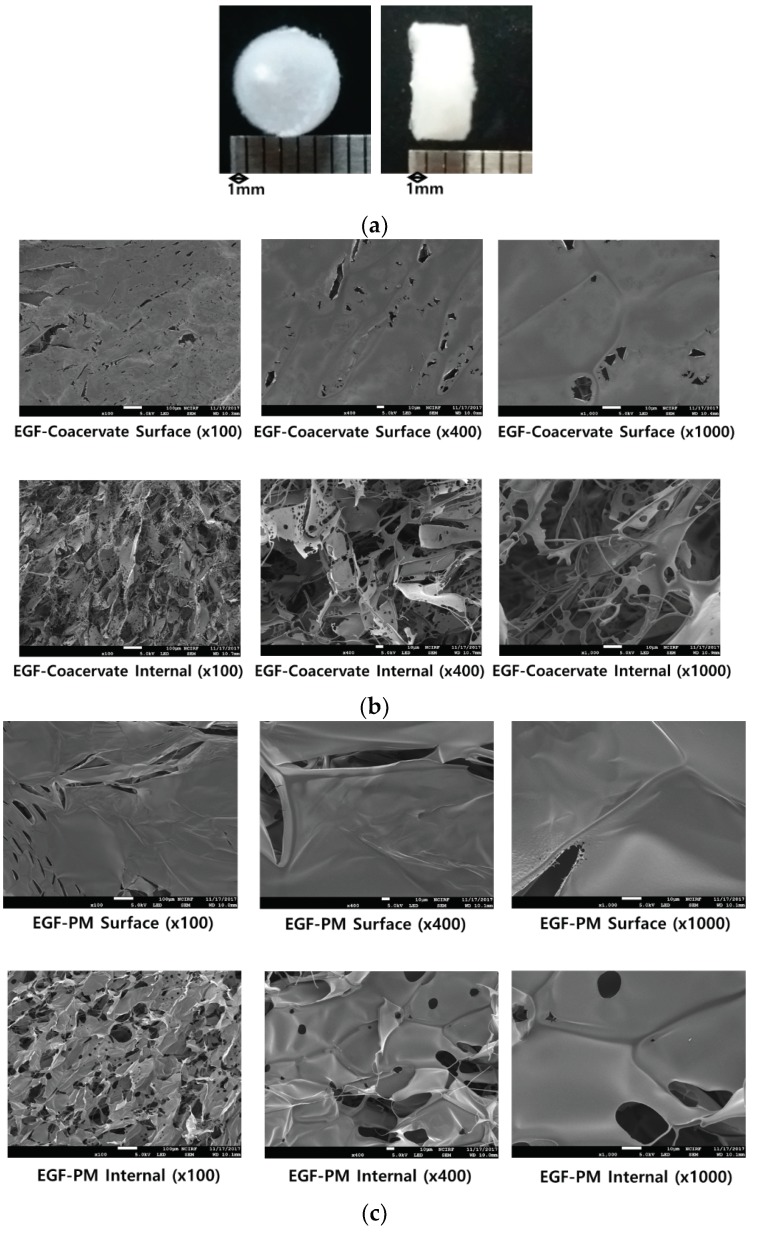
Morphology of freeze-dried coacervate and physical mixture. (**a**) Top and side view of freeze-dried, LWGA-SA (1:0.4), 8.2 mM acetic acid coacervate (Diameter: 7 mm, thickness: 3 mm). (**b**) SEM of the surface and internal structures of freeze-dried LWGA-SA (1:0.4), 8.2 mM acetic acid coacervate encapsulating EGF. Scale bars represent 100 μm (×100) and 10 μm (×400, ×1000). (**c**) SEM of the surface and internal structure of freeze-dried EGF-PM at various magnitudes. Scale bars represent 100 μm (×100) and 10 μm (×400, ×1000).

When freeze-dried, LWGA-SA (1:0.4) coacervates prepared at pH 4.34 became a white sponge (Figure 4a). The SEM data show that freeze-dried EGF-coacervates consisted of interconnected sheets with very fine pores and some porous fibers whereas EGF-PM appeared as inter-connected sheets of smooth texture with less number of pores than EGF-coacervates (Figure 4b,c). The freeze-dried coacervates returned to colloidal coacervates when it was hydrated in water whereas the freeze-dried EM appeared as solution upon hydration (data not shown). Freeze-drying of coacervates resulted in a sponge type structure which could be placed in the wound site and form a film upon hydration. Therefore, freeze-dried EGF coacervate would provide a novel delivery system with easy application to a wound site. 

### 3.5. Protection of EGF from Trypsin Digestion 

**Figure 5:**
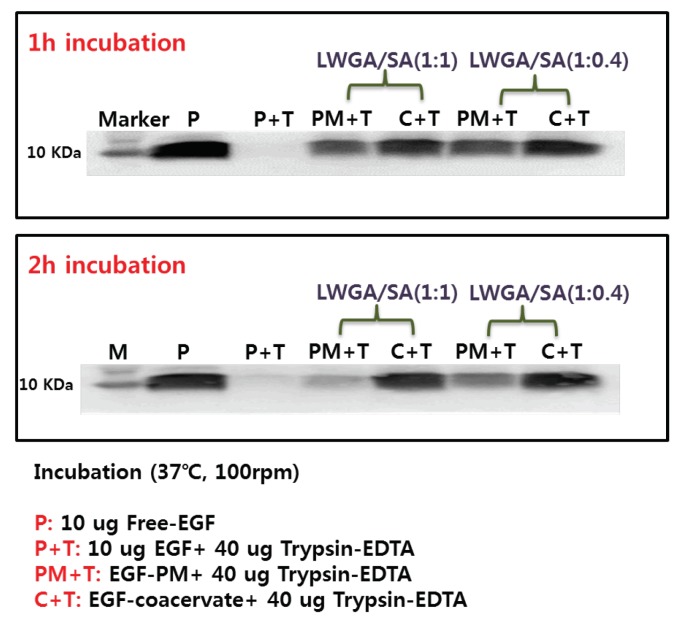
Trypsin digestion assay by Western blot. P: Free-EGF without trypsin, P+T: EGF solution with trypsin-EDTA, PM+T: EGF-PM with trypsin-EDTA, C+T: EGF-coacervates with trypsin-EDTA. The samples were incubated at 37 °C, 100 rpm for 1–2 h.

Among various compositions, two coacervate compositions with relatively high EGF encapsulation efficiency, LWGA-SA (1:1) at pH 4.14 and LWGA-SA (1:0.4) at pH 4.34, were selected to determine whether the encapsulated EGF in the coacervates could be protected from a protease. Trypsin was chosen as a model protease because it is a broad-spectrum serine protease. After 1 h incubation with 40 μg of trypsin-EDTA at 37 °C, EGF solution did not show any remaining EGF, indicating complete digestion by trypsin (Figure 5). The EGF band in EGF-PM became less dense in 2 h and EGF-PM composed of LWGA-SA (1:1) showed much less dense band than that of LWGA-SA (1:0.4). EGF in EGF-coacervate was visible even after 2 h for both polymer ratios, demonstrating that the present freeze-dried EGF-coacervate could be an advanced topical growth factor delivery system to circumvent proteases in the chronic wound bed. There are several previous reports on coacervate systems protecting encapsulated growth factors from trypsin digestion [7,8,30]. However, the LWGA-SA coacervate in the present study is, to the best of our knowledge, the first coacervate composed of natural polymers to effectively protect EGF from trypsin digestion. Notably, EGF in physical mixtures of gelatin and sodium alginate also showed slower digestion compared to free EGF solution and such effect was more prominent for LWGA-SA (1:0.4) than LWGA-SA (1:1) (Figure 5). Therefore, we speculate that gelatin itself acts as a competitor to trypsin digestion and protects EGF although not as effectively as coacervation does. Protection of EGF from protease upon application to chronic wound sites will contribute to enhanced activity of EGF. In order to corroborate the effect of encapsulation on EGF activity, in vitro activities of various samples on HDF were studied as below.

### 3.6. HDF Scratch Wound Assay

**Figure 6:**
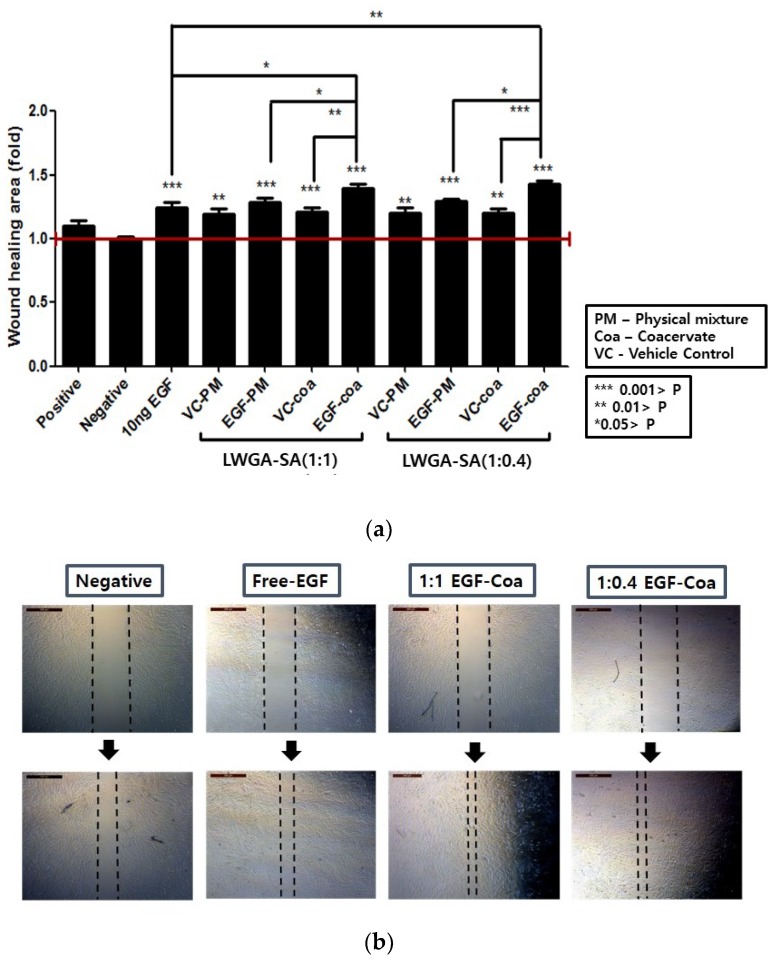
Human dermal fibroblast scratch wound assay. Positive control: 10% fetal bovine serum (FBS); Negative control: 0.5% FBS; Free-EGF (10 ng/mL solution); Freeze-dried samples (VC-PM) EGF-PM, VC-Coa, EGF-Coa). For each ratio of LWGA-SA, VC-PM stands for freeze dried samples of LWGA and SA solution, EGF-PM for freeze dried sample of LWGA, SA, and EGF in solution, VC-Coa for freeze dried sample without EGF, and EGF-Coa for freeze dried sample of LWGA-SA coacervates encapsulating EGF. (**a**) Quantitative wound healing rate by microscopic images analysis using Image-Pro Plus software, followed by one way ANOVA with GraphPad Prism software for statistical analysis. (**b**) Representative microscopic images of scratch wounds on confluent HDFs monolayers after treatment with media alone, free EGF solution, EGF-Coa with LWGA-SA (1:1), and EGF-Coa with LWGA-SA (1:0.4): Upper panels at time 0 and lower panels at 8 h time point of incubation.

HDF scratch wound assay has been frequently used to evaluate wound healing [30,40]. EGF solution exhibited a significant migratory effect in HDFs at 10 ng/mL compared to the negative control (Figure 6). Under the same EGF concentration, both LWGA-SA (1:1) and (1:0.4) EGF-coacervates (EGF-Coa) were more effective in enhancing migration of HDFs than EGF solution. EGF-Coa were also more effective than freeze-dried samples of LWGA and SA in solution (VC-PM), the physical mixture of LWGA, SA, and EGF in solution (EGF-PM), or coacervate of LWGA and SA without EGF (VC-Coa). All of them showed better migration activity of HDF than the negative control. The better activity of EGF-Coa than other samples is consistent with the trypsin digestion assay which demonstrated that encapsulation of EGF in coacervate effectively protected proteolysis of EGF relative to EGF-PM.

## 4. Conclusions

EGF incorporation in HWGA-SA precipitates and LWGA-SA coacervates at low pH supports our hypothesis that EGF would be encapsulated in GA-SA coacervates by electrostatic interactions between EGF and SA at pH lower than the pI of EGF. Encapsulation of EGF in coacervates effectively protected EGF from proteolysis and enhanced its in vitro activity in HDF. EGF-coacervates are proposed as a potential EGF delivery system to accelerate wound healing in chronic wounds such as DFU. EGF-coacervates in the present study have several advantages over other protein delivery systems. It is feasible to scale-up and is cost-effective because the manufacturing process involves simple mixing of aqueous solutions of LWGA, SA, and the protein of interest to encapsulate, followed by pH adjustment. It does not employ any synthetic polymers, organic solvent, high temperature, or chemical reactions and therefore, it is biocompatible and applicable to protein drugs. The characteristics of EGF-coacervates reported in the present study warrant further studies necessary for translation into clinical studies, such as evaluation of physico-chemical stability and in vivo efficacy for DFU. Different phase behaviors of GA-SA depending on the gelatin molecular weight are a further research subject for better comprehension of the polyelectrolyte complex. In particular, the contribution of H-bond interactions to early onset of precipitation in HWGA-SA reaction would be an interesting subject for future collaboration with researchers interested in theoretical approaches. Modulation of the present GA-SA coacervates to encapsulate other growth factors such as FGF or PDGF could also be an interesting future research subject for developing enhanced drug delivery systems for DFU.

## Figures and Tables

**Table 1:** pH dependence of the reaction between HWGA-SA and LWGA-SA at (1:0.4) polymer ratios.

HWGA-SA (1:0.4)							
pH	5.97 (0.01)	4.82 ^c^ (0.01)	4.55 ^φ^ (0)	4.36 (0.01)	4.26 (0)	4.17 (0.01)	4.09 (0)
Turbidity	0.03 (0)	1.68 (0.03)	2.09 (0)	2.00 (0)	1.99 (0)	1.90 (0)	1.73 (0)
Zeta potential	−51.60 (3.31)	−26.80 (0.2)	−30.07 (0.80)	−27.30 (0.50)	−25.50 (0.79)	−24.33 (1.39)	−23.47 (0.21)
Z-average (d.nm)	521.50 (101.76)	726.60 (79.29)	2130.67 (859.60)	3650.00 (115.21)	7481.00 (5542.93)	1474.33 (279.06)	2373.33 (844.92)
PDI	0.85 (0.051)	0.47 (0.035)	1 (0)	1 (0)	0.73 (0.24)	0.63 (0.322)	0.84 (0.273)
**LWGA-SA 1:0.4**							
pH	6.00 (0.01)	4.78 ^c^ (0.01)	4.52 (0.01)	4.34 (0)	4.25 ^φ^ (0.01)	4.14 (0)	4.07 (0)
Turbidity	0.03 (0)	1.32 (0)	1.91 (0)	2.01 (0)	1.97 (0)	1.88 (0)	1.69 (0)
Zeta potential	−34.97 (0.38)	−25.00 (0.26)	−24.37 (0.61)	−22.30 (0.56)	−22.27 (0.71)	−21.57 (1.51)	−16.47 (1.12)
Z-average (d.nm)	699.27 (62.57)	923.60 (25.91)	364.40 (10.89)	1191.00 (70.79)	2688.67 (476.82)	2422.67 (894.91)	1810.67 (123.87)
PDI	0.94 (0.098)	0.50 (0.021)	0.14 (0.016)	0.33 (0.025)	0.94 (0.098)	0.59 (0.267)	0.49 (0.116)

^c^: pH_c_, onset pH for coacervation at each LWGA-SA ratio. ^φ^: pH_φ_, onset pH for precipitation at each LWGA-SA ratio. *: Data are means and standard deviations (parenthesis) from triplicate measurements.

**Table 2:** Assignments of major bands in FTIR of HWGA, SA, and their mixtures (1:0.4) in various phases.

Peak Assignment	Amide III (C–N Stretching, N–H Deformation)	Amide II (C–N–H Deformation)	Amide I (C=O Stretching)	C–H Vibration	Amide B Stretching (–NH3+ Stretching)	COO–Stretching Vibration (C=O)	COOH (C=O Stretching)	Pyranose Ring (C–O–C Stretching)
HWGA powder	1236	1531	1633	~2937	~3076	N/A	N/A	N/A
SA powder	N/A	N/A	N/A	~2937	N/A	1595	1734	1211
Physical mixture of powder HWGA-SA	1236	split	*	No change	*	*	1734	1211
One phase solution HWGA-SA (pH 5.97)	1240	Triple split	Weak split	-	3076	-	-	-
Coacervates HWGA-SA (pH 4.82)	1240	Triple split	Weak split	-	3076	-	-	-
Precipitates HWGA-SA (pH 4.09)	Weak split	Quadruple split	Weak split	-	3076	-	-	-

*: Peak not detectable due to overlapping peaks in this region originating from HWGA and SA; -: Peak disappeared after reaction in solution, coacervation, or precipitation condition.

**Table 3:** Assignments of major bands in FTIR of LWGA, SA, and their mixtures (1:0.4) in various phases.

Peak Assignment	Amide III (C–N Stretching, N–H Deformation)	Amide II (C–N–H Deformation)	Amide I (C=O Stretching)	C–H Vibration	Amide B Stretching (–NH3+ Stretching)	COO–Stretching Vibration (C=O)	COOH (C=O Stretching)	Pyranose Ring (C–O–C Stretching)
LWGA powder	1236	1525	1635	~2937	~3070	N/A	N/A	N/A
SA powder	N/A	N/A	N/A	~2937	N/A	1595	1734	1211
Physical mixture of powder LWGA-SA	1240	Split	*	No change	*	*	1734	1211
One phase solution LWGA-SA (pH 5.97)	1238	Double Split	1635	No change	3078	-	1730	1203
Coacervates LWGA-SA (pH 4.82)	1238	Triple Split	Weak split	No change	3076	-	1724	-
Precipitates LWGA-SA (pH 4.09)	1238	Triple split	Weak split	No change	3078	-	-	-

*: Peak not detectable due to overlapping peaks in this region originating from LWGA and SA; -: Peak disappeared after reaction in solution, coacervation, or precipitation condition.

**Table 4:** Quantitative encapsulation efficiency and loading efficiency of EGF-coacervates.

GA:SA Ratio of EGF-Coa (Reaction pH)	Encapsulation Efficiency (%) Mean (SD) *n* = 3	Loading Efficiency (%) Mean (SD) *n* = 3
1:1 (4.14)	40.3 (1.03)	0.7 (0.02)
1:0.4 (4.34)	81.3 (0.35)	2.1 (0.01)

## Supplementary Materials

The following are available online at https://www.mdpi.com/1999-4923/11/10/530/s1, Table S1: pH dependence of the reaction between HWGA and SA at various polymer ratios. Table S2: pH dependence of the reaction between LWGA and SA at various polymer ratios.
