# Supplementary material for: Gelatin-Alginate Complexes for EGF Encapsulation: Effects of H-Bonding and Electrostatic Interactions
Source: Pharmaceutics. 2019 Oct 14;11(10):530. doi: 10.3390/pharmaceutics11100530 (PMC6835588; doi:10.3390/pharmaceutics11100530)
Supplement: Supplementary file 1 [file pharmaceutics-11-00530-s001.pdf]

pharmaceutics

MDPI

# Supplementary Materials: Gelatin-Alginate Complexes for EGF Encapsulation: Effects of H-Bonding and Electrostatic Interactions

**Seonghee Jeong, ByungWook Kim, Hui-Chong Lau and Aeri Kim**

**Table S1.** pH dependence of the reaction between HWGA and SA at various polymer ratios.

| | | | | | | | |
|---|---|---|---|---|---|---|---|
| **HWGA-SA (1:1)** | | | | | | | |
| pH | 6.23 (0.01) | 4.84 (0.02) | $4.58^{c}$ (0) | $4.42^{\varphi}$ (0.01) | 4.30 (0.01) | 4.21 (0.01) | 4.14 (0) |
| Turbidity | 0.05 (0) | 0.13 (0.01) | 1.62 (0.08) | 1.90 (0) | 1.97 (0) | 1.97 (0) | 1.91 (0) |
| Zeta potential | −52.73 (0.96) | −38.90 (1.14) | −39.37 (0.40) | −37.40 (0.70) | −36.63 (0.97) | −41.93 (1.07) | −39.47 (1.76) |
| Z-average (d.nm) | 563.10 (86.03) | 375.43 (55.00) | 722.53 (25.79) | 2315.33 (142.47) | 1114.67 (235.71) | 1158.87 (202.64) | 1272.67 (51.25) |
| PDI | 1 (0) | 0.98 (0.038) | 0.28 (0.078) | 0.65 (0.198) | 0.60 (0.095) | 0.58 (0.204) | 0.44 (0.13) |
| **HWGA-SA (1:0.8)** | | | | | | | |
| pH | 6.10 (0.01) | 4.83 (0.01) | $4.57^{c}$ (0.01) | $4.40^{\varphi}$ (0.01) | 4.29 (0.01) | 4.21 (0) | 4.14 (0.01) |
| Turbidity | 0.04 (0) | 0.12 (0.02) | 1.66 (0.04) | 1.93 (0) | 2.02 (0) | 1.99 (0) | 1.98 |
| Zeta potential | −52.70 (0.85) | −39.30 (1.04) | −37.03 (0.84) | −36.63 (1.40) | −33.63 (0.81) | −39.13 (0.76) | −38.03 (2.50) |
| Z-average (d.nm) | 551.77 (55.88) | 328.03 (47.99) | 641.07 (16.61) | 1090.00 (132.35) | 730.33 (41.44) | 921.73 (46.00) | 967.33 (48.19) |
| PDI | 1 (0.002) | 1 (0) | 0.18 (0.057) | 0.48 (0.118) | 0.42 (0.121) | 0.74 (0.245) | 0.55 (0.112) |
| **HWGA-SA (1:0.4)** | | | | | | | |
| pH | 5.97 (0.01) | $4.82^{c}$ (0.01) | $4.55^{\varphi}$ (0) | 4.36 (0.01) | 4.26 (0) | 4.17 (0.01) | 4.09 (0) |
| Turbidity | 0.03 (0) | 1.68 (0.03) | 2.09 (0) | 2.00 (0) | 1.99 (0) | 1.90 (0) | 1.73 (0) |
| Zeta potential | −51.60 (3.31) | −26.80 (0.2) | −30.07 (0.80) | −27.30 (0.50) | −25.50 (0.79) | −24.33 (1.39) | −23.47 (0.21) |
| Z-average (d.nm) | 521.50 (101.76) | 726.60 (79.29) | 2130.67 (859.60) | 3650.00 (115.21) | 7481.00 (5542.93) | 1474.33 (279.06) | 2373.33 (844.92) |
| PDI | 0.85 (0.051) | 0.47 (0.035) | 1 (0) | 1 (0) | 0.73 (0.24) | 0.63 (0.322) | 0.84 (0.273) |

$^{c}$: $pH_c$ , onset pH for coacervation at each HWGA-SA ratio. $^{\varphi}$: $pH_{\varphi}$, onset pH for precipitation at each HWGA-SA ratio. *Data are means and standard deviations (parenthesis) from triplicate measurements.

**Table S2.** pH dependence of the reaction between LWGA and SA at various polymer ratios.

| | | | | | | | |
|---|---|---|---|---|---|---|---|
| **LWGA-SA 1:1** | | | | | | | |
| pH | 6.00<br>(0) | 4.82<br>(0.01) | 4.55[c]<br>(0) | 4.38<br>(0.01) | 4.31<br>(0.06) | 4.22<br>(0.01) | 4.14<br>(0.01) |
| Turbidity | 0.04<br>(0) | 0.06<br>(0) | 1.25<br>(0) | 1.78<br>(0.01) | 1.87<br>(0) | 1.90<br>(0) | 1.94<br>(0) |
| Zeta potential | −42.13<br>(1.08) | −35.70<br>(0.44) | −36.23<br>(0.67) | −37.30<br>(0.61) | −35.80<br>(2.26) | −36.63<br>(1.90) | −35.70<br>(1.31) |
| Z-average (d.nm) | 334.17<br>(24.07) | 527.27<br>(26.15) | 864.17<br>(19.15) | 807.87<br>(10.74) | 639.17<br>(11.79) | 955.00<br>(41.35) | 914.13<br>(20.08) |
| PDI | 0.95<br>(0.067) | 0.77<br>(0.207) | 0.37<br>(0.013) | 0.31<br>(0.032) | 0.21<br>(0.019) | 0.39<br>(0.033) | 0.30<br>(0.05) |
| **LWGA-SA 1:0.8** | | | | | | | |
| pH | 6.06<br>(0.02) | 4.82<br>(0.01) | 4.53[c]<br>(0.01) | 4.39<br>(0.01) | 4.27<br>(0.01) | 4.20[φ]<br>(0.01) | 4.12<br>(0.01) |
| Turbidity | 0.04<br>(0) | 0.07<br>(0) | 1.43<br>(0) | 1.82<br>(0) | 1.86<br>(0) | 1.88<br>(0) | 1.97<br>(0) |
| Zeta potential | −41.30<br>(0.56) | −36.80<br>(1.15) | −36.40<br>(0.90) | −34.23<br>(0.65) | −35.90<br>(0.78) | −25.53<br>(0.81) | −22.87<br>(0.68) |
| Z-average (d.nm) | 497.80<br>(101.31) | 693.13<br>(17.63) | 673.27<br>(1.55) | 601.33<br>(3.87) | 659.93<br>(5.70) | 1112.23<br>(196.67) | 5348.67<br>(2912.65) |
| PDI | 0.75<br>(0.065) | 0.68<br>(0.05) | 0.30<br>(0.036) | 0.25<br>(0.015) | 0.21<br>(0.033) | 0.49<br>(0.065) | 0.40<br>(0.048) |
| **LWGA-SA 1:0.4** | | | | | | | |
| pH | 6.00<br>(0.01) | 4.78[c]<br>(0.01) | 4.52<br>(0.01) | 4.34<br>(0) | 4.25[φ]<br>(0.01) | 4.14<br>(0) | 4.07<br>(0) |
| Turbidity | 0.03<br>(0) | 1.32<br>(0) | 1.91<br>(0) | 2.01<br>(0) | 1.97<br>(0) | 1.88<br>(0) | 1.69<br>(0) |
| Zeta potential | −34.97<br>(0.38) | −25.00<br>(0.26) | −24.37<br>(0.61) | −22.30<br>(0.56) | −22.27<br>(0.71) | −21.57<br>(1.51) | −16.47<br>(1.12) |
| Z-average (d.nm) | 699.27<br>(62.57) | 923.60<br>(25.91) | 364.40<br>(10.89) | 1191.00<br>(70.79) | 2688.67<br>(476.82) | 2422.67<br>(894.91) | 1810.67<br>(123.87) |
| PDI | 0.94<br>(0.098) | 0.50<br>(0.021) | 0.14<br>(0.016) | 0.33<br>(0.025) | 0.94<br>(0.098) | 0.59<br>(0.267) | 0.49<br>(0.116) |

[c]: $pH_c$ , onset pH for coacervation at each LWGA-SA ratio. [φ]: $pH_{\varphi}$, onset pH for precipitation at each LWGA-SA ratio. *Data are means and standard deviations (parenthesis) from triplicate measurements.
